# Overexpression of LPCAT1 enhances endometrial cancer stemness and metastasis by changing lipid components and activating TGF-β/Smad2/3 signaling pathway: Tumor-promoting effect of LPCAT1 in endometrial cancer

**DOI:** 10.3724/abbs.2022076

**Published:** 2022-07-06

**Authors:** Tianyi Zhao, Rui Sun, Xiaohong Ma, Lina Wei, Yixin Hou, Kun Song, Jie Jiang

**Affiliations:** Department of Gynecology and Obstetrics Qilu Hospital Cheeloo College of Medicine Shandong University Jinan 250012 China

**Keywords:** endometrial cancer, lipid, LPCAT1, stemness, TGF-β/Smad2/3 signaling pathway

## Abstract

The incidence of endometrial cancer (EC) increases annually and tends to occur in younger women. A particularly important relationship exists between EC and metabolic disorders. As one of the most important components of lipid metabolism, phospholipids play an indispensable role in metabolic balance. LPCAT1 is a key enzyme regulating phospholipid metabolism. In this study, we perform further investigations to seek mechanistic insight of LPCAT1 in EC. Our results demonstrate that silencing of LPCAT1 inhibits the growth of endometrial cancer, while overexpression of LPCAT1 results in enhanced stemness and metastasis in endometrial cancer cell lines. Meanwhile, the contents of various phospholipids including phosphatidylethanolamine (PE), phosphatidylcholine (PC), and triglyceride (TG) change significantly after overexpression of LPCAT1. In addition, through RNA-sequencing and western blot analysis, we observe that the TGF-β/Smad2/3 signaling pathway is of great importance in the tumor-promoting function of LPCAT1. LPCAT1 promotes the expressions of stem cell-related transcription factors and epithelial-mesenchymal transition (EMT) related proteins through the TGF-β/Smad2/3 signaling pathway. Moreover, we find that TSI-01, which can inhibit the activity of LPCAT1, is able to restrain the proliferation of EC cell lines and promote cell apoptosis. Collectively, we demonstrate that LPCAT1 enhances the stemness and metastasis of EC by activating the TGF-β/Smad2/3 signaling pathway and that TSI-01 may have potential use for the treatment of EC.

## Introduction

As the second most prevalent pelvic gynecologic malignancy worldwide and the most prevalent pelvic gynecologic malignancy in the USA and Europe, endometrial cancer (EC) affects and claims the lives of many women [ 1, 2] . EC often develops in women with concurrent metabolic syndrome and obesity [3], and the incidence of EC has increased in parallel with the growing rates of obesity [ 4– 6] . Previous studies showed that a 5-unit increase in body mass index (BMI) is linked to a >50% increase in the risk of developing EC [3]. Almost one in three of the world’s population was obese or overweight in 2016 and the number of EC patients will continue to increase over the next few years [ 7, 8] . Meanwhile, previous studies on EC mainly focused on the levels of gene alterations [ 9, 10] . It remains to be clarified how metabolic disorders affect the pathogenesis of EC.

Altered lipid metabolism is considered to be one of the hallmark features of cancers [11]. Lysophosphatidylcholine acyltransferases (LPCATs) are of great importance in regulating intracellular lipid metabolism and homeostasis [ 12– 14] . LPCAT1, as a key member of the LPCAT family, has been demonstrated to play an important role in different tumor types [15]. Increased copy number and expression of LPCAT1 have been found in breast cancer, acute myeloid leukemia, and colorectal cancer and are correlated with a poor prognosis [ 16– 18] . In lung adenocarcinoma, LPCAT1 has been shown to activate the PI3K/AKT/MYC pathway, thereby promoting brain metastasis of cancer cells [19]. It has also been reported that phospholipid remodeling of the cell membrane mediated by LPCAT1 drives continuous activation of the cell growth factor signaling pathway and promotes glioblastoma progression [20]. Moreover, Du *et al*. [21] found an up-regulation of LPCAT1 in clear cell renal cell carcinoma (ccRCC) tissues and demonstrated that LPCAT1 promotes ccRCC progression probably by transferring lysophosphatidylcholine (LPC) into phosphatidylcholine (PC). A growing amount of evidence suggests that lipid metabolism, regulated by LPCAT1, is widely implicated in tumor progression [ 20, 22, 23] . However, the role of LPCAT1 in EC, which is closely related to metabolic disorders, has rarely been studied.

In the present study, on the basis of our previous study [24], we demonstrate that silencing of LPCAT1 inhibits the growth of EC, while overexpression of LPCAT1 results in enhanced stemness and metastasis in EC cell lines. The transforming growth factor-beta (TGF-β)/mothers against decapentaplegic homolog2/3 (Smad2/3) signaling pathway is essential to the function of LPCAT1. Moreover, changes in lipid metabolites may be an intermediate factor between LPCAT1 and the malignant phenotype. Finally, TSI-01 is found to be a potential drug targeting LPCAT1.

## Materials and Methods

### Cell culture and chemicals

Human EC cell lines Ishikawa, HEC-1A, RL-95, and AN3CA were obtained from Zhong Qiao Xin Zhou Biotechnology Co., Ltd (Shanghai, China), and KLE cells were purchased from Procell Life Science & Technology Co., Ltd (Wuhan, China). All the above cell lines were recently authenticated by using short tandem repeat (STR) analysis. Ishikawa cells were maintained in Dulbecco’s modified Eagle’s medium (DMEM; BI, Beit-Hamek, Israel). HEC-1A cells were cultured in McCoy’s 5A medium (Gibco, Carlsbad, USA) with 1% sodium pyruvate solution. RL-95 cells were cultured in DMEM-F12 (Gibco) with 1% sodium pyruvate solution and 5 μg/mL insulin. AN3CA cells were cultured in Minimal Essential Medium (MEM; Gibco) with 1% sodium pyruvate solution and 1% non-essential amino acid solution. KLE cells were cultured in DMEM-F12. All the media contained 10% fetal bovine serum (v/v) and 1% penicillin-streptomycin (v/v), and all the cells were cultured in an incubator containing 5% CO _2_ at 37°C. The compound TSI-01 was purchased from APExBio Company (Houston, USA).

### Clinical samples and materials

Pathology-confirmed tumor tissues were obtained from 89 endometrioid adenocarcinoma patients and normal endometrium tissues from 27 patients who had a hysterectomy owing to other benign diseases. All patients underwent surgery in the Department of Obstetrics and Gynecology of Qilu Hospital of Shandong University from 2014 to 2020. All experiments in this study were approved by the Ethics Committee of the Qilu Hospital of Shandong University.

### The Cancer Genome Atlas (TCGA) data acquisition and bioinformatics

The EC gene expression data and clinical phenotype information were downloaded from TCGA. Molecular subtype information was downloaded from the Uterine Corpus Endometrial Carcinoma (TCGA, PanCancer Atlas) dataset of cBioPortal. The one-class logistic regression (OCLR) algorithm was used to calculate the stemness index based on gene expression profiles of EC patients [25].

### Immunohistochemical (IHC) staining

All clinical specimens were fixed in formalin and embedded in paraffin, and 4-μm sections were cut, dewaxed, hydrated, and antigen-retrieved with sodium citrate (pH 6.0). After blocking with endogenous peroxidase, serum albumin blocking was performed to retrieve antigens for the non-specific binding sites. Rabbit anti-LPCAT1 antibody (Cat. No. ab214034; Abcam, Cambridge, UK) was added to the tissue sections and incubated at 4°C for 12–16 h. Incubation with a secondary antibody was performed using horseradish peroxidase (HRP)-labeled goat anti-mouse antibody for 30 min at room temperature (RT), followed by treatment with diaminobenzidine (DAB) for 1 min. Hematoxylin was added to the tissue sections for 5 min at RT. For quantitative IHC analysis, plaque images were visualized and analyzed with a microscopic imaging analysis system (IX71; Olympus, Tokyo, Japan). IHC staining was scored for both positive cell proportion (0 score: 0%, 1 score:<25%, 2 score: 25%–50%, 3 score: 51%–75%, and 4 score: ≥ 75%) and staining intensity (0 score: negative, 1 score: weak, 2 score: moderate, and 3 score: strong), which ultimately resulted in designations of complete loss of expression, or weak, moderate, or strong expression, respectively.

### RNA interference and lentivirus transfection

For further investigation, we compared the expression of LPCAT1 in different cell lines. The expression level was the highest in the Ishikawa cell line and the lowest in the HEC-1A cell line. Therefore, we choose the Ishikawa cell line for knockdown experiment verification, and the HEC-1A cell line for overexpression verification. Small interfering RNA (siRNA) for LPCAT1 and negative control siRNA were designed and synthesized by GenePharma (Shanghai, China). We first selected three interfering RNAs to test their efficiency at the protein level. Finally we selected siLPCAT1-550 (sense 5′-CCAUGACGAUGUCCUCCAUTT-3′, antisense 5′-AUGGAGGACAUCGUCAUGGTT-3′) to establish knock-down cell lines. Ishikawa cells were transfected with LPCAT1 siRNA and control siRNA (sense 5′-UUCUCCGAACGUGUCACGUTT-3′, antisense 5′-ACGUGACACGUUCGGAGAATT-3′) for 4 h using Lipofectamine 3000 in Opti-MEM (Invitrogen, Carlsbad, USA). To establish stable cell lines, lentivirus was purchased from GeneChem (Shanghai, China). HEC-1A cells were infected with the pUbi-LPCAT1 and pUbi-Ctrl lentivirus. After 48 h, cells were screened with 2 μg/mL puromycin (Solarbio, Beijing, China) for 1–2 weeks to obtain stable cell lines.

### Western blot analysis

Cells were harvested at the logarithmic growth stage, washed three times with phosphate buffered saline (PBS; pre-cooled at 4°C) and lysed using radioimmunoprecipitation assay lysis buffer (Beyotime, Haimen, China) supplemented with phenylmethanesulfonyl fluoride (PMSF) and phosphatase inhibitor at a ratio of 100:1:1 for 30 min on ice. The lysates were centrifuged at 13,000 *g* for 15 min at 4°C. Then a bicinchoninic acid (BCA) protein assay kit (Tiangen Biotech Co., Ltd, Beijing, China) was used to detect the protein concentration. Loading buffer was added to the supernatant and the mixture was incubated for 5 min at 100°C. Protein extracts (30–50 μg) were separated by 10% sodium dodecyl sulfate/polyacrylamide gel electrophoresis (SDS-PAGE), transferred onto a 0.22-μm polyvinylidene fluoride membrane and blocked with 5% skimmed milk at RT for 1 h. The membranes were then incubated with primary antibodies at 4°C overnight on a shaker, followed by wash with 1× Tris-buffered saline containing Tween (TBST). Then membranes were incubated with HRP-linked secondary antibodies (Cell Signaling Technology, Beverly, USA) at RT for 1 h. HRP Substrate Reagent (Thermo Fisher Scientific, Waltham, USA) was used to detect protein bands using Image Quant LAS 4000 (General Electric Company, Boston, USA). Finally, the bands were quantified using ImageJ software. The antibodies against the following proteins were used: β-actin (#4970; 1:1000; Cell Signaling Technology, Beverly, USA), LPCAT1 (ab214034; 1:2000; Abcam), Smad (#8685; 1:1000; Cell Signaling Technology), p-Smad (#8828; 1:1000; Cell Signaling Technology), TGF-β1 (sc-130348; 1:1000; Santa Cruz, Santa Cruz, USA), Nanog (sc-293121; 1:1000; Santa Cruz), SRY-box transcription factor 2 (SOX2) (sc-365823; 1:1000; Santa Cruz), organic cation/carnitine transporter 3/4 (Oct-3/4) (sc-5279; 1:1000; Santa Cruz), antigen identified by monoclonal antibody Ki-67 (ki-67) (ab92742; 1:1000; Abcam), vimentin (#5741; 1:1000; Cell Signaling Technology), cyclinB1 (sc-166210; 1:1000; Santa Cruz), and zonula occludens-1 (ZO-1) (#8193; 1:1000; Cell Signaling Technology).

### RNA isolation and qRT-PCR assay

Total RNA was extracted using Trizol reagent (Invitrogen), concentration and purity were detected using a spectrophotometer (Thermo Fisher Scientific). Subsequently, the RNA was transcribed into cDNA. qRT-PCR was carried out to quantify RNA expression using the SYBR Green Master Mix (TaKaRa, Dalian, China) on a StepOne™ PCR amplifier (Applied Biosystems, Foster City, USA). Primers used included (5′ to 3′): *β*- *actin*-F: GAAGAGCTACGAGCTGCCTGA; *β*- *actin*-R: CAGACAGCACTGTGTTGGCG; and *LPCAT1*-F: GGCGGAACCCCTTCGTG; *LPCAT1*-R: GGGAAGAGCGTCAGTGTCAT.

### Colony formation assay and sphere-forming assay

After transfection with siRNA or lentivirus, Ishikawa and HEC-1A cells (2 mL) were seeded at a concentration of 200 cells/mL and 300 cells/mL, respectively, in each well of 6-well plates. After incubation at 37°C for 14 days, cells were fixed with 4% paraformaldehyde for 15 min and stained with crystal violet (Beyotime) at RT for 30 min. Images were captured under a microscope and then analyzed using ImageJ software to count the number of colonies.

Sphere cultures were established by seeding 2000 cells into each well of 24-well low-attachment-plates (Corning Life Sciences, Corning, USA) containing serum-free DMEM-F12 medium, 2% B27 (Thermo Fisher Scientific), epidermal growth factor (20 ng/mL; Thermo Fisher Scientific), basic fibroblast growth factor (10 ng/mL, Thermo Fisher Scientific), and 4 μg/mL insulin (Sigma, St Louis, USA). After incubation for two weeks, secondary sphere culture was performed by enzymatic dissociation and then further cultured for two weeks. The sphere forming efficiency was observed under a microscopic imaging analysis system (IX71).

### CCK-8 and 5-ethynyl-20-deoxyuridine (EdU) assay

Transfected cells were seeded in 96-well plates at 2×10 ^3^ cells per well for Ishikawa cells and 3×10 ^3^ cells per well for HEC-1A cells. Cells were incubated for 1–5 days at 37°C. Aliquots of 10 μL of Cell Counting Kit-8 (CCK-8; Zhongshan Golden Bridge, Beijing, China) reagent were added into each well and incubated at 37°C for 1 h. The absorbance of each well was measured at 450 nm and recorded to calculate the cell proliferation activity.

The proliferation activity of cells was also determined using an EdU incorporation assay kit (RiboBio, Guangzhou, China). The assay was performed according to the manufacturer’s protocol. The transfected Ishikawa and HEC-1A cells were seeded in 96-well plates at 6×10 ^3^ cells per well, and were cultured in an incubator at 37°C overnight. Aliquots of 50 μM of EdU were added into each well and incubated for 2 h. Cell fluorescence was captured using a fluorescence microscope (Olympus) and analyzed using ImageJ software.

### Cell apoptosis assay

Transfected cells were harvested at the logarithmic growth phase using trypsin without ethylenediaminetetraacetic acid (EDTA), gently washed three times with PBS, and stained using a fluorescein isothiocyanate (FITC) Annexin V Apoptosis Detection kit (BD Bioscience Pharmingen, San Diego, USA) according to the manufacturer’s protocol. Cells were incubated for 30 min at RT and separated by fluorescence-activated cell sorting (FACS) flow cytometry on a BD flow cytometer (BD Biosciences, Franklin Lakes, USA). The data were analyzed with FlowJo v10 software.

### Cell cycle assay

Cells were harvested when the cell density reached ~70%, washed three times with PBS and fixed with 99% ethanol solution (pre-cooled at –20°C) at 4°C overnight. The next day, the cells were rewashed with PBS, and then stained with propidium iodide (BD Biosciences) according to the manufacturer’s protocol. The cell cycle distribution was detected by FACS flow cytometry and analyzed with Modifit LT software.

### Transwell assay

Transwell assays were performed using Transwell chambers (Corning Life Sciences) coated with or without Matrigel (BD Biosciences; 1:9 dilution). A total of 2×10 ^5^ transfected cells suspended in medium without serum were added to the upper chamber. Medium containing 10% fetal bovine serum (FBS; 700 μL) was added to the lower chamber. After incubation for 20–24 h, cells in the upper chamber were gently removed using a cotton swab, and cells on the transwell membrane were fixed with methanol and stained with hematoxylin. Images were captured with a microscopic imaging analysis system (IX71). The number of migrated or invaded cells was counted in three random fields at a magnification of 200×.

### Wound healing assay

A total of 1×10 ^6^ transfected cells were seeded in each well of 6-well plates, incubated overnight, and scratch wounds were generated using 10-μL pipette tips. The cell debris was washed away and cells were incubated in 1% FBS medium. Images of wound healing process were captured at the same position at 0 and 72 h. The area that healed at 72 h relative to 0 h was used to calculate the cell migration activity.

### Tumor xenograft model

Female athymic BALB/c nude mice were purchased from Beijing Vital River Laboratory Animal Technology Co., Ltd (Beijing, China) and maintained in a pathogen-free facility. The mice were treated in accordance with protocols approved by the Ethical Committee of Shandong University. Stable transfected HEC-1A cells (1×10 ^7^) were mixed with 100 μL of PBS and injected subcutaneously into the right armpit of each 5-week-old mouse. Mouse body weights and tumor sizes were measured every other day from day 5 after injection. Tumor volumes were calculated as: V = [(length × width ^2^)/2]. The tumor mass was weighed after the mice were sacrificed. qRT-PCR, western blot analysis and IHC were used to measure the expression levels of target molecules.

### RNA sequencing assay

The HEC-1A cells stably infected with pUbi-LPCAT1 and pUbi-Ctrl lentivirus were used to identify differentially expressed mRNAs. Three samples of each group were disrupted with Trizol reagent (Invitrogen) and sent to Origingene Bio-pharmaceutical Technology Co., Ltd (Shanghai, China) for mRNA sequencing. The libraries were created using an Illumina TruSeq ^TM^ RNA sample prep kit (Illumina Inc., San Diego, USA). Processed RNA-sequencing data were analyzed and bioinformatics analysis was performed. Genes with |logFC|>1 and *P* value<0.05 were considered as significantly differentially expressed genes.

### Cell sample preparation and extraction

HEC-1A cells stably infected with the pUbi-LPCAT1 and pUbi-Ctrl lentivirus were collected and placed in liquid nitrogen for 2 min, then the samples were thawed and vortexed. The above steps were repeated three times and the samples were then centrifuged at 4°C at 2 300 *g* for 1 min. Subsequently, samples were homogenized with 1 mL of mixture which contained methyl tert-butyl ether (MTBE) and internal standard mixture, and were vortexed for 15 min. Then 200 μL water was added and vortexed for 1 min. The samples were centrifuged at 13,000 *g* for 10 min. Finally, the supernatant was dissolved in mobile phase B and stored at –80°C for liquid chromatography (LC)-electrospray ionization (ESI)-tandem mass spectrometry (MS/MS) analysis.

### High performance liquid chromatography (HPLC)

The LC-ESI-MS/MS system [Ultra Performance Liquid Chromatography (UPLC), ExionLC AD′ https://sciex.com.cn/; MS, QTRAP® System, https://sciex.com/] was used to analyze sample extracts. The separation system was an Accucore™ C30 column (2.6 μm, 2.1 mm ×100 mm; Thermo Fisher Scientific) with temperature set at 45°C. Mobile phase A was an aqueous 60% acetonitrile solution and mobile phase B was an isopropanol 10% acetonitrile isopropanol solution. The gradient program was set with following ratio and time point: 80/20 (V/V) at 0 min, 70/30 (V/V) at 2 min, 40/60 (V/V) at 4 min, 15/85 (V/V) at 9 min, 10/90 (V/V) at 14 min, 5/95 (V/V) at 15.5 min, 5/95 (V/V) at 17.3 min, 80/20 (V/V) at 17.5 min, 80/20 (V/V) at 20 min. Aliquots of 2 μL sample were injected into the column at a flow-rate of 0.35 mL/min. The effluent was hooked up to an ESI-triple quadrupolar linear ion trap (QTRAP)-MS.

### ESI-QTRAP-MS/MS

A triple quadrupole-linear ion trap mass spectrometer (QTRAP), QTRAP® LC-ESI-MS/MS System, was used to collect UPLC information. The ESI source operation parameters were as follows: ion source; turbo spray; source temperature 500°C; ion spray (IS) voltage 5500 V (positive), –4500 V (negative); ion source gas 1 (GS1), gas 2 (GS2), and curtain gas (CUR) were set at 45, 55, and 35 psi, respectively; the collision gas (CAD) was medium. There were both positive and negative ionic modes under Linear Ion Trap (LIT) and triple quadrupole (QQQ) acquisitions. The overall scanning process was monitored by the analysis software 1.6.3 (SCIEX).

### Statistical analysis and bioinformatics analysis

The data were analyzed and mapped using GraphPad Prism 8.0 software (San Diego, USA). The Shapiro-Wilk normality test was used to examine the normal distribution of data. Metabolites with absolute log _2_FC ≥1 and variable importance in projection (VIP) ≥1 were considered significantly regulated. VIP values which contain permutation plots and score plots were extracted from orthogonal partial least-squares discriminant analysis (OPLS-DA) results. Statistical significance was determined using Student’s *t*-test, the Mann-Whitney U-test, and analysis of variance (ANOVA). Differences at *P*<0.05 were considered significant for all statistical tests.

### Data availability

The endometrial cancer gene expression data and clinical phenotype information can be downloaded from The Cancer Genome Atlas (TCGA). The counts data of RNA-seq are provided in Supplementary Material.

## Results

### LPCAT1 is upregulated in EC and the upregulation of LPCAT1 is associated with poor prognosis of EC

**Figure 1:**
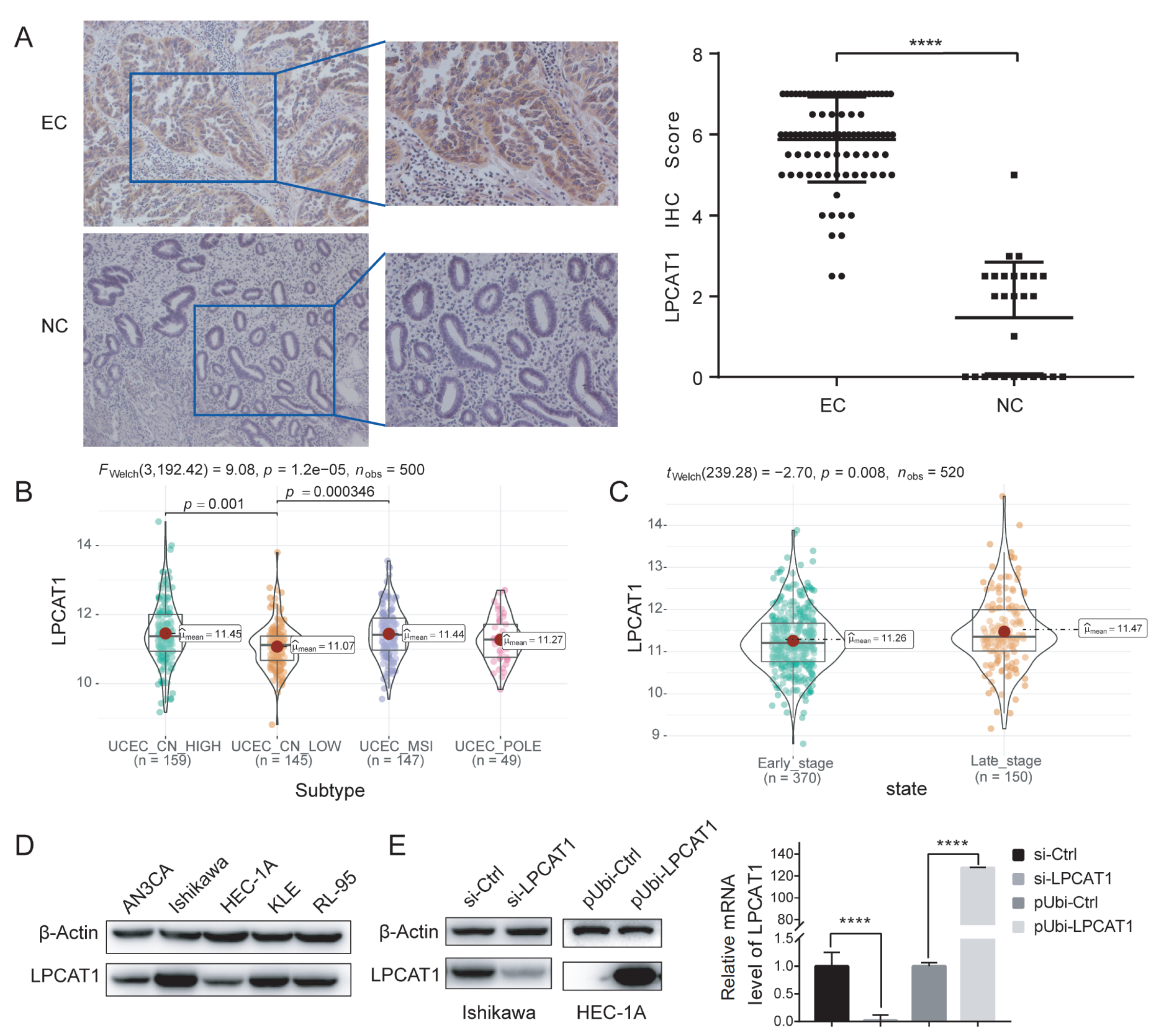
Figure 1 Upregulation of LPCAT1 is associated with poor prognosis of EC (A) Representative images of IHC staining of LPCAT1 in endometrial cancer tissues (left) and normal endometrium tissues (right). (B) The mRNA level of LPCAT1 in different EC TCGA molecular subtypes of the TCGA-UCEC cohort. (C) The mRNA level of LPCAT1 in different EC stages of the TCGA-UCEC cohort. (D) Western blot analysis was used to detect the expression of LPCAT1 in different EC cell lines. (E) PCR and western blot analysis were used to verify the efficiency of LPCAT1 si-RNA and the overexpression of lentivirus in Ishikawa and HEC-1A cell lines, respectively. Mann–Whitney U-test and Welch’s t test was used for statistical analysis. **** P<0.0001.

Our previous study demonstrated that LPCAT1 might serve as a molecular marker to predict the prognosis and status of the EC microenvironment and it is negatively associated with patient’s survival [24]. In the present study, we further confirmed these findings by collecting a greater number of patient specimens for IHC staining. Our results showed that, compared with that in normal tissues, the expression of LPCAT1 was significantly increased in EC tissues ( Figure 1A). According to TCGA data analysis, 500 EC patients were divided into four molecular subtypes, including microsatellite instability hypermutated (MSI) subtype, copy-number high (CN-HIGH) subtype, copy-number low (CN-LOW) subtype, and *POLE*mut subtype. It is known that EC patients diagnosed with CN-HIGH and MSI have a poorer prognosis than other molecular subtypes [ 26, 27] . We found that the expression of LPCAT1 in patients with MSI and CN-HIGH subtypes was significantly higher than that in the copy-number low (CN-LOW) subtype ( *P*<0.01; Figure 1B). Meanwhile, there was no obvious difference between the *POLE*mut subtype and other subtypes. Moreover, 520 EC patients were divided into two clinical stages: the early-stage patients included stage I, stage IA, stage IB, stage IC, stage II, stage IIA, and stage IIB; the late-stage patients included stage III, stage IIIA, stage IIIB, stage IIIC, stage IIIC1, stage IIIC2, stage IV, stage IVA, and stage IVB. We found that the late-stage patients tended to have high LPCAT1 expression ( Figure 1C). These findings suggest that the upregulation of LPCAT1 is associated with poor prognosis of EC.

### Selection of cell lines and establishment of transfected cell lines

For further investigation, we compared the expressions of LPCAT1 in different EC cell lines. Our results showed the highest expression in the Ishikawa cell line, and the lowest expression in the HEC-1A cell line ( Figure 1D). To investigate the biological function of LPCAT1 in EC, we firstly used si-RNA to silence *LPCAT1* in the Ishikawa cell line. Three interfering RNAs were used to test their efficiency at the protein and RNA levels ( Figure 1E), and si-LPCAT1-550 was chosen to establish knock-down cell lines. Meanwhile, the HEC-1A cell line was chosen for verification of lentivirus overexpression. The efficiency of overexpression was validated at the protein and RNA levels ( Figure 1E).

### Loss of LPCAT1 inhibits EC cell proliferation and increases cell apoptosis *in vitro*

**Figure 2:**
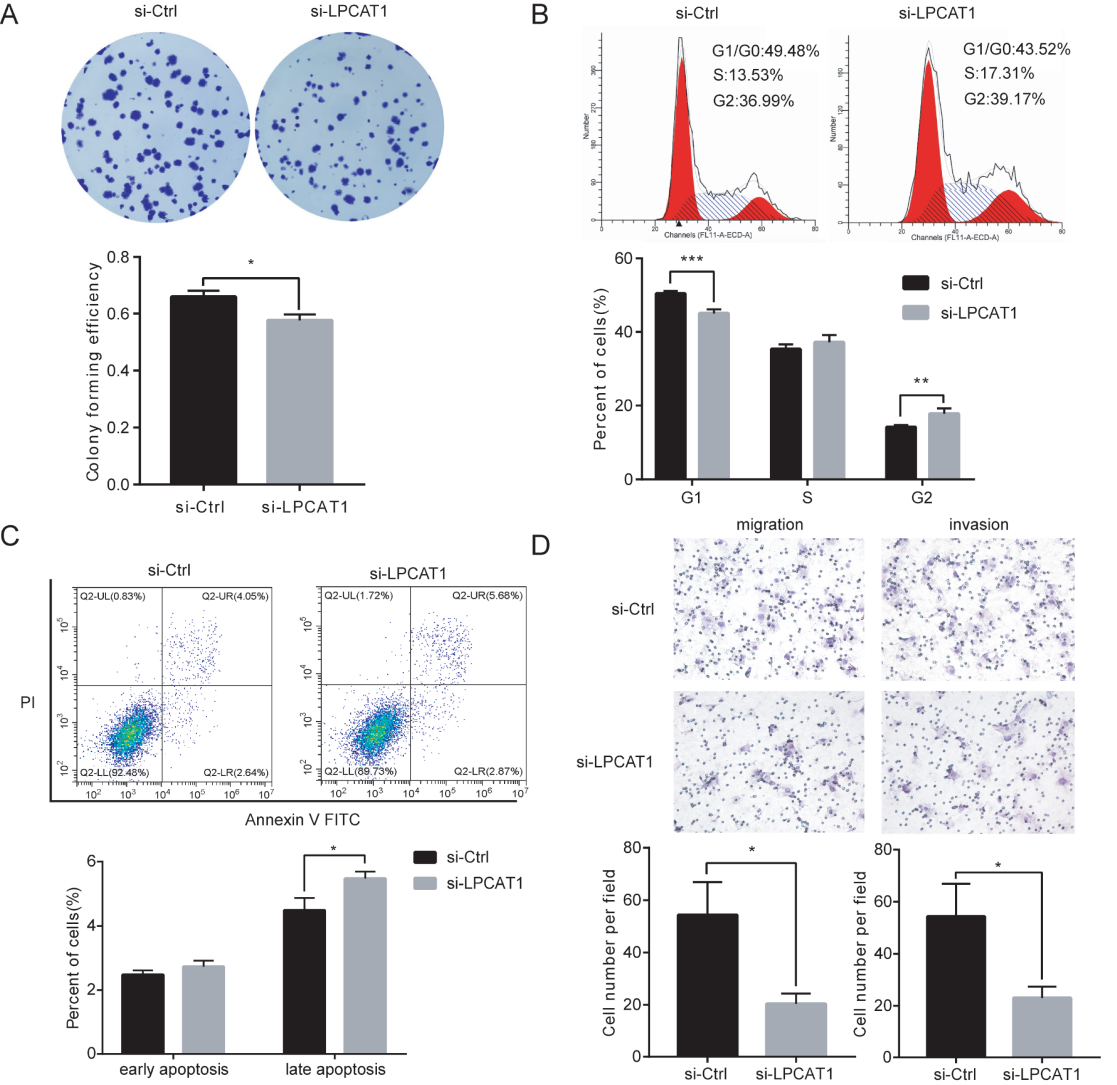
Figure 2 Loss of LPCAT1 inhibits EC cell proliferation and increases cell apoptosis *in vitro* (A) The colony formation ability of Ishikawa cells was assessed by colony formation assay. (B) Cell-cycle analysis was performed to determine the effect of LPCAT1 on the cell-cycle of Ishikawa cells. (C) Apoptosis assay showed that the down-regulation of LPCAT1 expression led to an increase in the apoptosis rate of cells. (D) Transwell assays were performed to detect the effect of LPCAT1 on the invasion and migration ability of Ishikawa cells. Student’s t-test was used for statistical analysis. * P<0.05, ** P<0.01, *** P<0.001.

**Figure 4:**
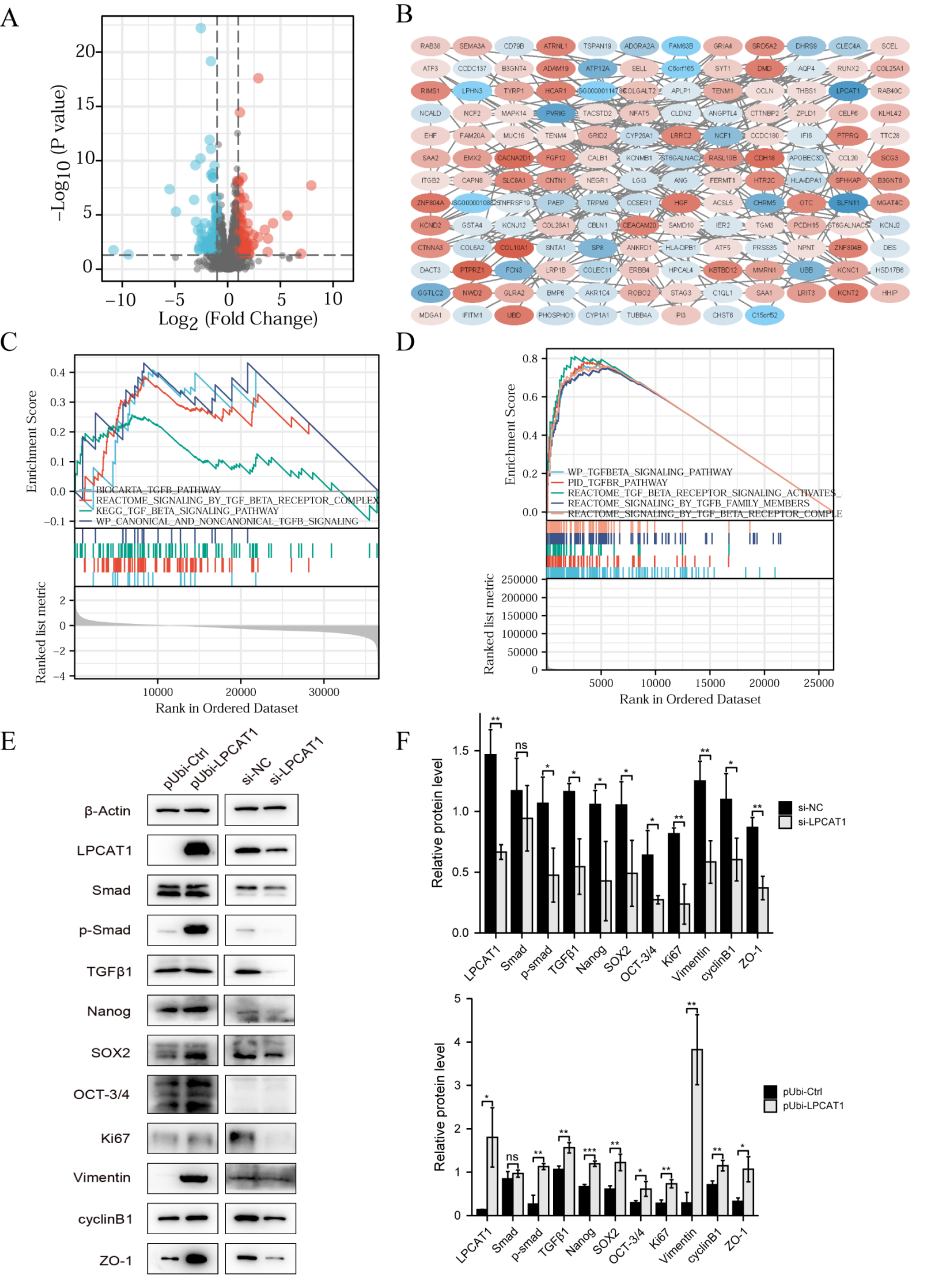
Figure 4 Overexpression of LPCAT1 activates the TGF-β signaling pathway (A) Volcano plot of differentially expressed genes. (B) Protein-protein interaction (PPI) network of differentially expressed genes. (C) TGF-β-related pathways enriched by GSEA between low and high LPCAT1 expression groups in TCGA-UCEC. (D) GSEA-enriched pathways between pUbi-LPCAT1 and pUbi-Ctrl HEC-1A cells. (E) Western blot analysis was used to examine the protein expressions of the TGF-β/Smad2/3 signaling pathway and related phenotype. (F) Quantification of the western blots shown in E using ImageJ software. Student’s t-test was used for statistical analysis. * P<0.05, ** P<0.01, *** P<0.001.

After verification of the interference efficiency of si-RNA, si-LPCAT1 and si-Ctrl Ishikawa cells were harvested after three days of infection for the subsequent assays. In colony formation experiments, si-LPCAT1 suppressed the colony formation ability of Ishikawa cells. The number and volume of clonal cell communities were both decreased in si-LPCAT1 cells ( Figure 2A). The percentage of cells in each cell cycle phase was measured by flow cytometry. Compared with the control cells, the down-regulation of LPCAT1 expression resulted in G2/M phase arrest ( Figure 2B). According to cell-cycle analysis, we detected the protein expression of CyclinB1, a marker of the G2/M phase. It was found that CyclinB1 expression was decreased after down-regulation of LPCAT1 expression ( Figure 4E). The results of apoptosis assay showed that the down-regulation of LPCAT1 led to an increase in the apoptosis rate of cells ( Figure 2C). Transwell assay was performed to detect the function of LPCAT1 in cell migration and invasion. After downregulation of LPCAT1 expression, the invasion and migration ability of Ishikawa cells was decreased ( Figure 2D).

### Stable over expression of LPCAT1 promotes EC cell stemness and migration ability *in vitro*

**Figure 3:**
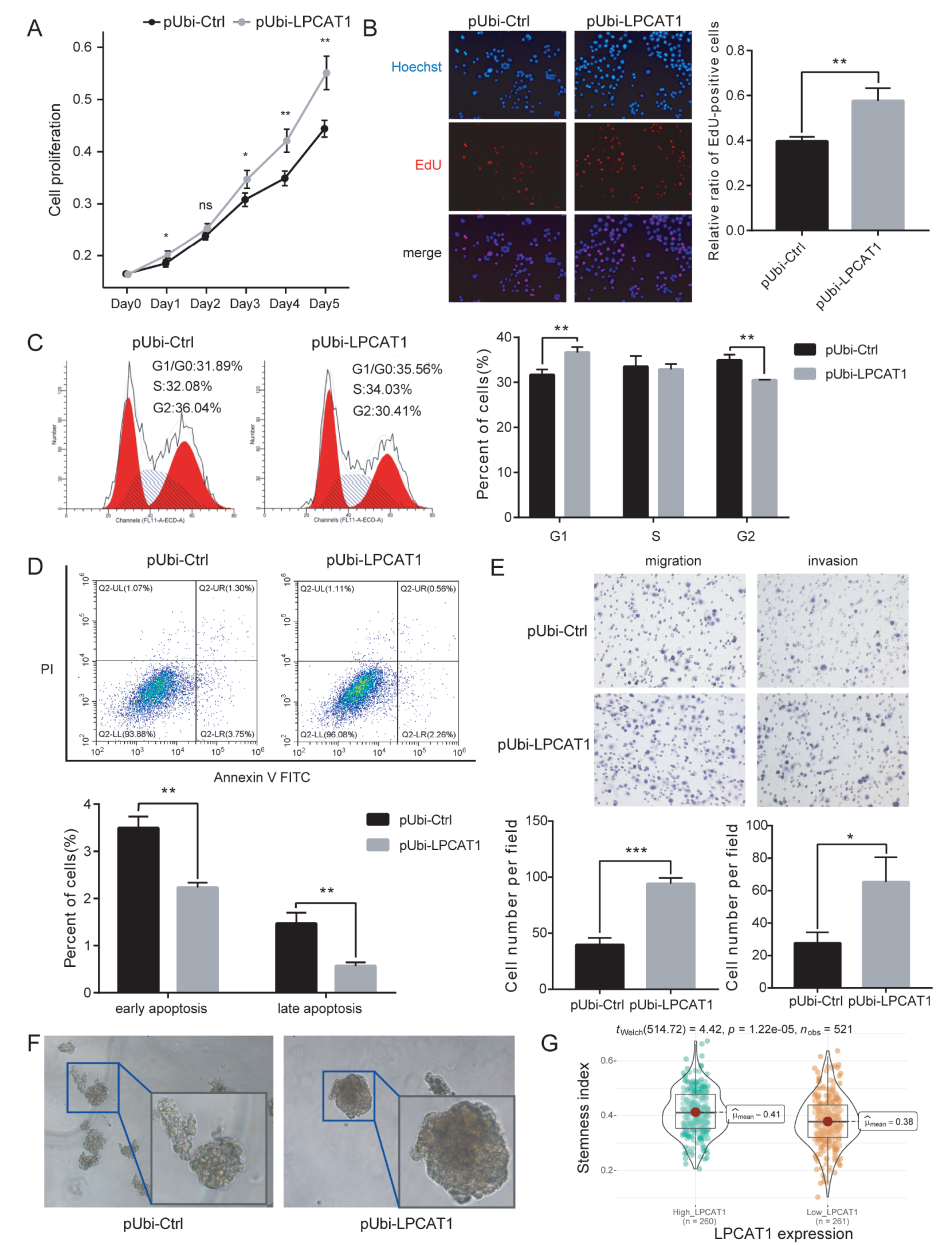
Figure 3 Overexpression of LPCAT1 promotes EC cell proliferation and decreases cell apoptosis *in vitro* (A) CCK-8 assay was used to detect the proliferation of pUbi-LPCAT1 and pUbi-Ctrl HEC-1A cells. (B) The cell proliferation ability of pUbi-LPCAT1 and pUbi-Ctrl HEC-1A cells was determined by EdU assay. (C) Cell-cycle analysis was performed to determine the effect of LPCAT1 overexpression on the cell-cycle of HEC-1A cells. (D) Apoptosis assay showed that the up-regulation of LPCAT1 expression led to a decrease in the apoptosis rate of cells. (E) Transwell assay was performed to detect the effect of LPCAT1 overexpression on the invasion and migration ability of HEC-1A cells. (F) The sphere formation assay of pUbi-LPCAT1 and pUbi-Ctrl HEC-1A cells for a two-week incubation. (G) The stemness index of patients in TCGA cohort according to their LPCAT1 expression. Student’s t-test and Welch’s t test was used for statistical analysis. * P<0.05, ** P<0.01, *** P<0.001.

To evaluate the functional role of LPCAT1, the HEC-1A cell line was stably transfected with the pUbi-LPCAT1 lentivirus. After verification of the transfection efficiency, pUbi-LPCAT1 and pUbi-Ctrl cells were harvested for the subsequent assays. Growth curve analysis demonstrated that up-regulation of LPCAT1 significantly increased cell growth compared with that in the control group ( Figure 3A). EdU assay also suggested that LPCAT1 increased cell growth ability ( Figure 3B). In cell cycle experiments, up-regulating the expression of LPCAT1was found to promote cells to enter the next cell cycle from G2/M phase ( Figure 3C). At the same time, CyclinB1 was up-regulated in pUbi-LPCAT1 HEC-1A cells, which was consistent with the cell cycle assay ( Figure 4E). Furthermore, the apoptosis assay showed that LPCAT1 over expression protected EC cells and reduced the rate of cell apoptosis ( Figure 3D). The transwell assay showed that overexpression of LPCAT1 enhanced the migration and invasive abilities of HEC-1A cells ( Figure 3E). The sphere formation assay showed that the upregulation of LPCAT1 expression caused an increase in the size of spheres, suggesting that LPCAT1 may promote the stemness of EC cells ( Figure 3F). According to TCGA data analysis, 521 EC patients were divided into LPCAT1 high expression group and LPCAT1 low expression group. Then we calculated the Spearman correlations between the stemness signature of them, and found that the stemness index was significantly higher in the LPCAT1 high expression group than that in the LPCAT1 low expression group ( Figure 3G).

### Overexpression of LPCAT1 activates the TGF-β/Smad2/3 signaling pathway

We next explored the potential signaling pathway regulated by LPCAT1. By RNA-sequencing analysis, we identified significantly differentially expressed genes (DEGs) between pUbi-LPCAT1 and pUbi-Ctrl HEC-1A cells ( Figure 4A) and analyzed the interaction among them ( Figure 4B). Moreover, all the samples in TCGA-UCEC cohorts were divided into low and high groups based on the median expression of LPCAT1 and gene set enrichment analysis (GSEA) was conducted to identify pathways between low and high LPCAT1 expression groups. The results showed that pathways related to TGF-β were significantly enriched ( Figure 4C). Meanwhile, we also perform GSEA analysis in DEGs between pUbi-LPCAT1 and pUbi-Ctrl HEC-1A cells and genes were enriched in pathways principally related to TGF-β ( Figure 4D). Moreover, western blot analysis results showed that silencing of *LPCAT1* reduced the expression levels of phosphorylated Samd2/3, while overexpression of LPCAT1 resulted in significantly upregulated phosphorylated Samd2/3 ( Figure 4E,F).

### Stable overexpression of LPCAT1 promotes EC cell tumorigenesis *in vivo* and TSI-01 is shown to inhibit the growth of EC cell lines

**Figure 5:**
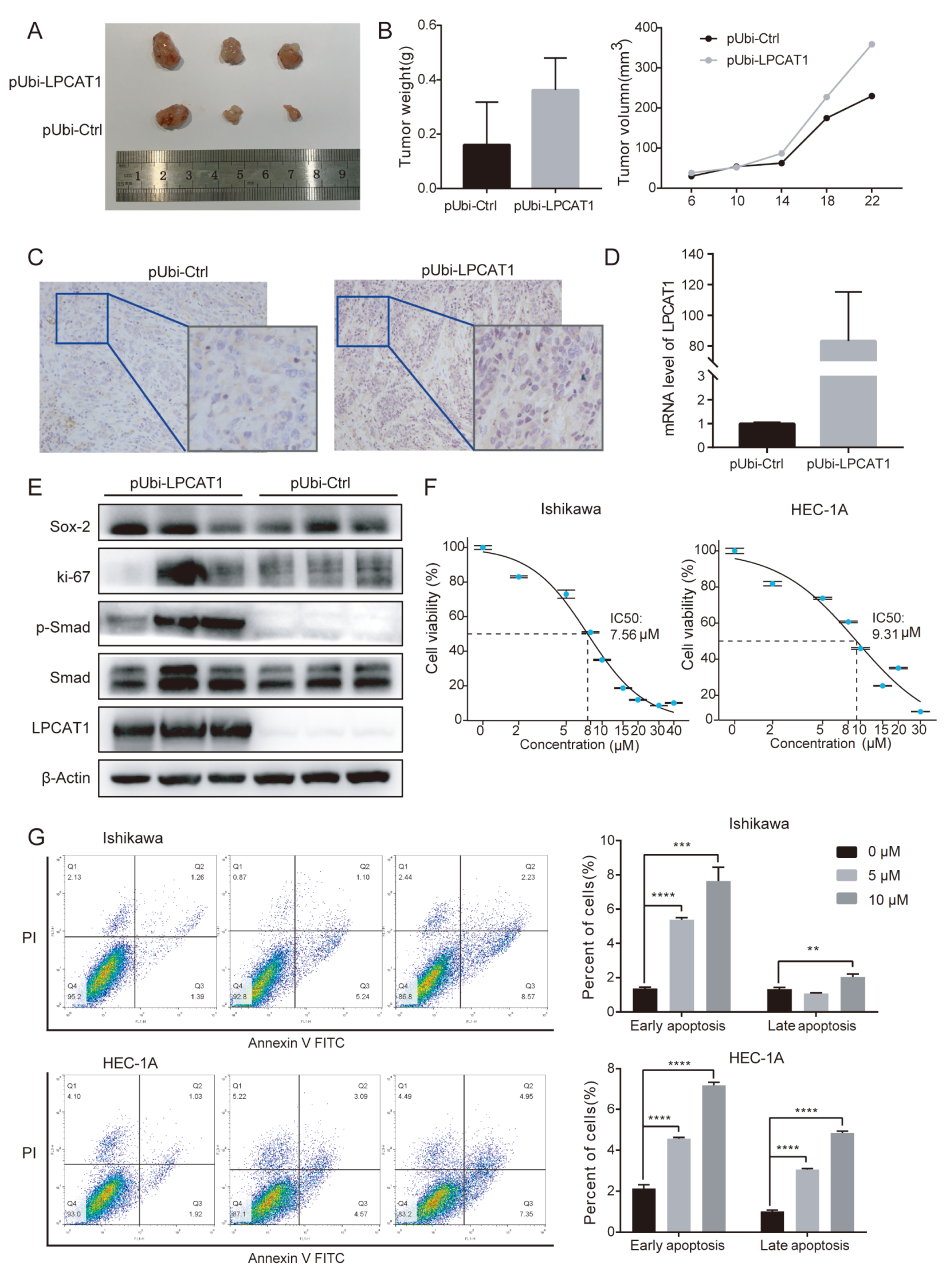
Figure 5 Overexpression of LPCAT1 promotes EC cell tumorigenesis *in vivo* and TSI-01 inhibites EC cell proliferation (A) Tumor xenograft formation of pUbi-LPCAT1 and pUbi-Ctrl HEC-1A cells, with each group containing three mice. (B) The tumor weight at the time of mouse sacrifice, and the tumor volume growth curve after injection with pUbi-LPCAT1 and pUbi-Ctrl HEC-1A cells. (C) The tumor sections were subject to IHC staining using antibodies against LPCAT1. (D) The mRNA level of LPCAT1 in tumor sections. (E) The protein levels of LPCAT1, Smad, p-Smad, ki-67, and SOX2 in tumor sections. (F) The IC 50 of TSI-01 in Ishikawa and HEC-1A cell lines detected by CCK-8 assay. (G) Apoptosis assay showed an increase in the apoptosis rate of cells with the increase of TSI-01 concentration. Student’s t-test was used for statistical analysis. ** P<0.01, *** P<0.001, **** P<0.0001.

We established mouse xenograft tumor models to investigate whether elevated LPCAT1 expression affects tumor growth *in vivo*. PUbi-LPCAT1 HEC-1A cells and pUbi-Ctrl HEC-1A cells were injected into left and right armpits of female BALB/c nude mice to generate xenograft models, respectively. The tumor volumes of mice injected with pUbi-LPCAT1 HEC-1A cells were significantly greater than those in the pUbi-Ctrl group ( Figure 5A). The same conclusion was drawn from the tumor weigh and the tumor volume growth curve ( Figure 5B). A portion of the tumor mass was embedded in paraffin and cut into 4-μm sections for IHC experiments ( Figure 5C). The remainder of the tumor mass was isolated either for qRT-PCR experiments to verify LPCAT1 expression ( Figure 5D), or for western blot analysis experiments to verify LPCAT1, Smad, p-Smad, ki-67, and SOX2 expressions ( Figure 5E). The compound TSI-01 was reported to inhibit the enzyme activity of LPCAT1 [28]. To further explore whether TSI-01 can affect the malignant phenotype of EC cells, we first detected the IC _50_ value of TSI-01 in Ishikawa and HEC-1A cell lines using the CCK-8 assay. The results showed that the IC _50_ of TSI-01 in the Ishikawa cell line was 7.56 μM, and the IC _50_ of TSI-01 in the HEC-1A cell line was 9.31 μM ( Figure 5F). Next, we conducted apoptosis experiments using 5 μM and 10 μM TSI-01, and found that the amount of apoptotic cells increased notably with the increase in the concentration of TSI-01 ( Figure 5G).

### Screening of differential lipid metabolites regulated by LPCAT1 and Kyoto Encyclopedia of Genes and Genomes (KEGG) substance classification of differential lipids.

**Figure 6:**
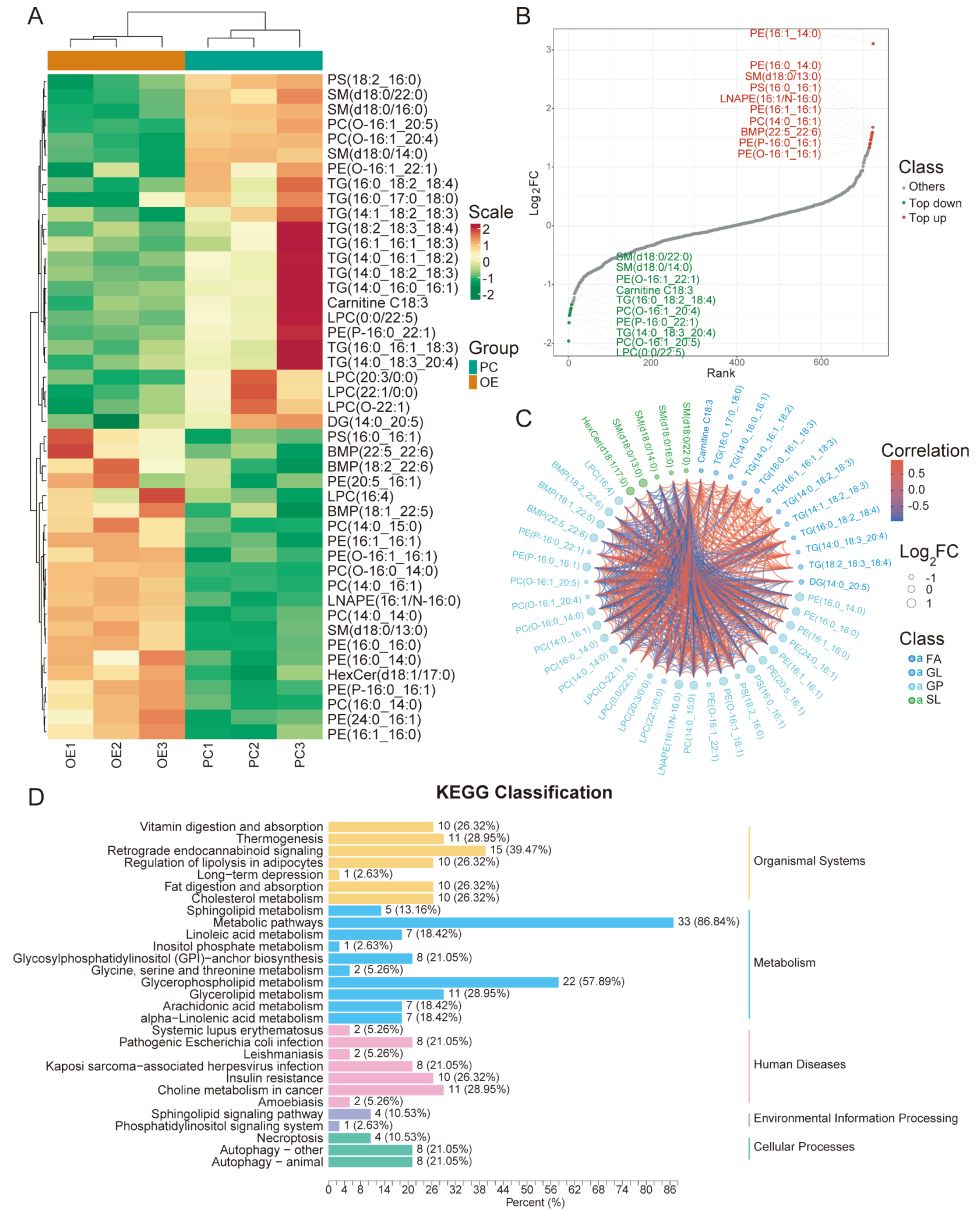
Figure 6 The correlation and KEGG substance classification of differential lipid metabolites regulated by LPCAT1 (A) Heatmap of differential lipid metabolites (PC represents pUbi-Ctrl, OE represents pUbi-LPCAT1). (B) Dynamic distribution diagram of differential lipid metabolites. Top 10 up-regulated lipid metabolites are shown in red and the top 10 down-regulated lipid metabolites are shown in green. (C) Chord diagram shows the correlation among differential lipid metabolites. (D) KEGG classification of differential lipid metabolites.

To explore the lipid metabolic alterations mediated by LPCAT1, a broad-spectrum lipid metabolome was collected. As expected, compared with the pUbi-Ctrl group, the pUbi-LPCAT1 group had a different lipid composition. In total, 45 significantly differential lipid metabolites were screened with 24 lipid metabolites being down-regulated and 21 lipid metabolites up-regulated ( Figure 6A). According to FC values, the top 10 up-regulated and top 10 down-regulated lipid metabolites were visualized in Figure 6B. The significantly up-regulated lipid metabolites were mainly phosphatidylethanolamine (PE) and PC. The significantly down-regulated lipid metabolites were mainly LPC and triglyceride (TG). The correlation among significant differential lipid metabolites is shown in the chord diagram in Figure 6C. To explore the potential pathways influenced by differential lipid metabolites, KEGG classification analysis was carried out. The results showed that two human disease pathways, insulin resistance and choline metabolism in cancers, involve most of the differential lipid metabolites ( Figure 6D).

## Discussion

The morbidity and disease-related mortality of EC which is the most common gynecological malignancy in Europe and North America is increasing annually [ 29, 30] . Although many studies have reported that malignant behaviors of EC are exacerbated by aberrant high expressions of genes [ 31, 32] , the mechanisms involving metabolism alteration and EC stemness need urgent clarification. In this study, we verified that high expression of LPCAT1 is associated with endometrial cancer. Moreover, we verified that LPCAT1 expression is correlated with TCGA molecular classification. The expression of LPCAT1 is higher in the TCGA molecular subtypes of EC with poorer prognosis. Silencing of LPCAT1 inhibits EC cell proliferation, stemness, migration, invasion, and promotes cell apoptosis in EC cell lines. Furthermore, overexpression of LPCAT1 could promote cell proliferation, stemness, migration, invasion, and reduce cell apoptosis in EC cells. Through RNA sequencing and experimental verification, we found that the TGF-β/Smad2/3 signaling pathway is essential for the biological functions of LPCAT1. Furthermore, TSI-01 may inhibit the progression of EC by targeting LPCAT1.

LPCAT1 level is highly elevated and may play a potential oncogenic role in multiple human cancers [ 33– 35] . For example, the expression of LPCAT1 is significantly upregulated in newly diagnosed acute myeloid leukemia (AML) cases compared with healthy controls, and thus may serve as an independent prognostic and diagnostic indicator for AML [17]. Uehara *et al*. [36] found that LPCAT1 expression level was highly upregulated in gastric cancer lesions compared to the level in nonneoplastic mucosa. Based on our previous study, we further demonstrated that the expression of LPCAT1 was upregulated in EC with more IHC histologic sections, which is consistent with that of AML and gastric cancer. Moreover, LCPAT1 expression is correlated with TCGA molecular classification, and the expression of LPCAT1 is higher in those subtypes with poorer prognosis. It has been well acknowledged that proliferation, migration, dedifferentiation, and invasiveness play an important role in metastasis and tumorigenesis [37]. Morita *et al*. [23] demonstrated that *LPCAT1* serves as a tumor-promoting gene that promotes tumor proliferation and metastasis in hepatocellular carcinoma. Additionally, *LPCAT1* plays a pro-tumorigenic role in tumor development and progression in prostate cancer [38] and oral squamous cell carcinoma [39]. In the present study, we found that silencing of *LPCAT1* inhibited endometrial cancer cell colony formation, stemness, migration, and invasion; and promoted cell apoptosis and arrested EC cells at the G2/M phase of the cell cycle. Additionally, overexpression of LPCAT1 also promoted endometrial cancer growth *in vivo*. Taken together, our results suggest that LPCAT1 plays a tumor-promoting role and the silencing of *LPCAT1* inhibits cell stemness and metastasis in EC, similar to the role of LPCAT1 in hepatocellular carcinoma [23] and oral squamous cell carcinoma [39].

TGF-β signaling pathway plays a dual role in tumor development and metastasis [ 40– 42] . It shows two completely opposite responses in the process of tumor development: either inhibiting or promoting tumorigenesis. In normal or precancerous cells, TGF-β signaling pathway acts as a tumor suppressor [41]. Meanwhile, with the development of a tumor, cancer cells can selectively bypass inhibition of the TGF-β signaling pathway and give full play to the tumor promoting effect of the TGF signaling pathway [42]. TGF-β/Samd2/3 signaling pathway has been demonstrated to participate in the proliferation, stemness, migration, and invasion of various cancer cells [43]. A previous study indicated that the TGF-β signaling pathway is implicated in malignant phenotypes in human EC [44], and activation of the TGF-β/Smad2/3 signaling pathway could enhance endometrial cancer stemness [45]. We found that silencing of *LPCAT1* could inhibit the TGF-β/Smad2/3 signaling pathway in EC cell lines; conversely, further study demonstrated that overexpression of LPCAT1 could activate the same pathway. Taken together, this evidence suggests that LPCAT1 serves as an oncogene and the TGF-β/Smad2/3 signaling pathway is of crucial importance in LPCAT1-mediated development of tumors.

LPCAT1 is a key phospholipid metabolic enzyme which catalyzes the synthesis of PC from LPC [46]. Several studies have shown that the conversion from LPC to PC, catalyzed by LPCAT1, is responsible for tumor progression [ 21, 23] . In this study, we found that overexpression of LPCAT1 resulted in increased cellular PC and PE and reduced cellular LPC and TG through UPLC-MS/MS lipidomics detection. LPC has been regarded as an antitumor lipid that could regulate immune response [47]. This may partly explain the pro-tumor effects of LPCAT1. Studies have shown that targeting the PE biosynthesis pathway improves the efficacy of chemotherapy in liver cancer [48]. In addition, abundant PE is responsible for the colonization of lung cancer and lack of PE would inhibit the proliferation of lung cancer [49]. Our study shows that LPCAT1 can clearly activate the TGF-β/Smad2/3 signaling pathway. However, it is unclear which modalities work, either LPCAT1 directly binds to a molecule in the pathway or a certain type of lipid plays a part, which is the limitation of this study.

**Figure 7:**
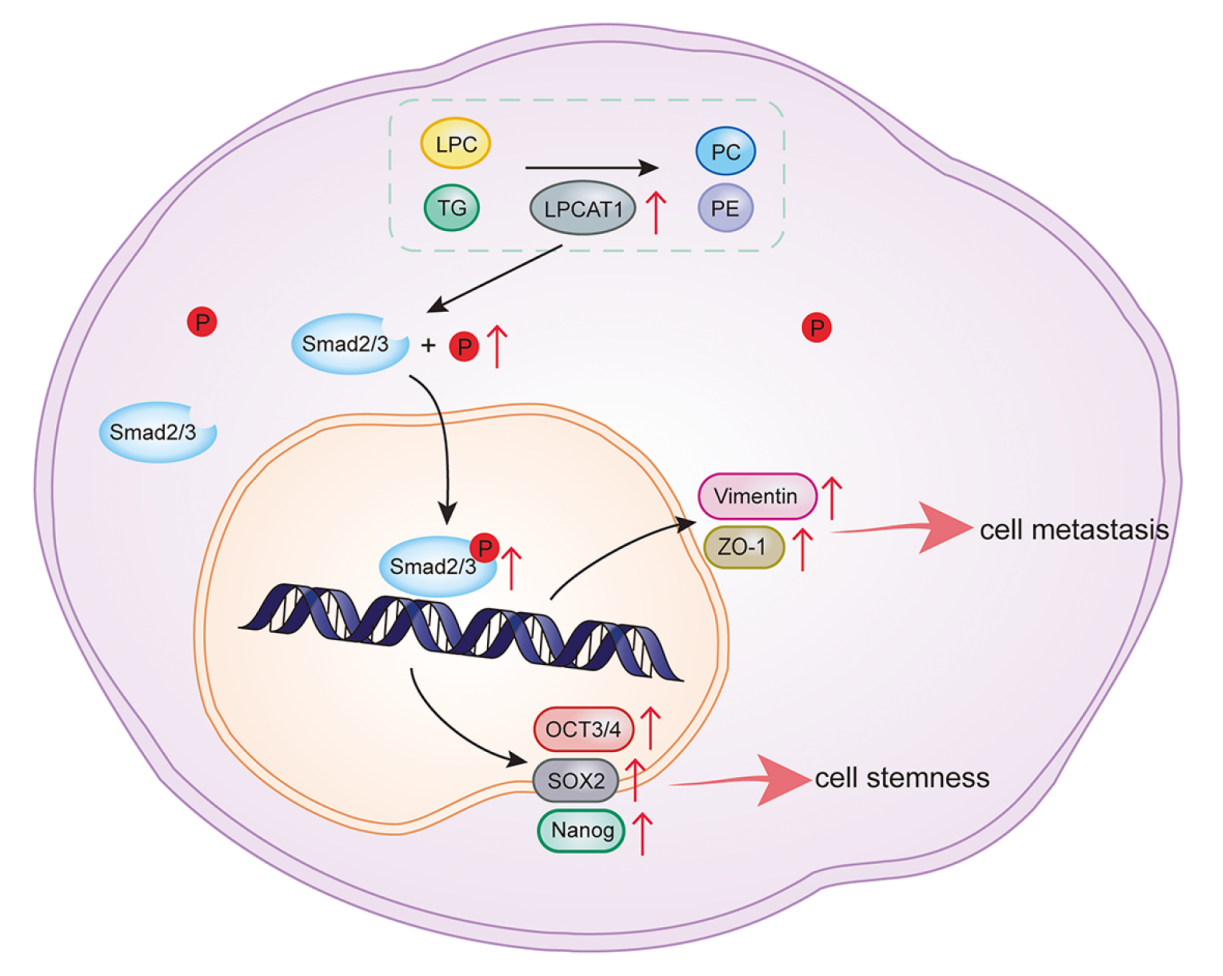
Figure 7 Schematic model of LPCAT1-mediated stemness and metastasis of endometrial cancer The overexpression of LPCAT1 can promote the stemness and metastasis of EC through activating the TGF/β-Smad2/3 signaling pathway and changing lipid components.

In summary, we demonstrated that LPCAT1 is upregulated in EC samples and overexpression of LPCAT1 promotes proliferation, metastasis, cell stemness, and reduces cell apoptosis by activating the TGF-β/Smad2/3 pathway ( Figure 7). Moreover, LPCAT1 significantly changes cellular lipid components. PC and PE are significantly increased while TG and LPC are significantly decreased in stable LPCAT1-overexpressing cell lines. These results provide, to our knowledge, the first evidence of a modulated relationship between LPCAT1 and the TGF-β/Samd2/3 pathway. However, the exact relationship among LPCAT1, lipid alterations, and the TGF-β/Samd2/3 pathway is still unknown. More experiments need to be conducted to further explore the exact relationship between LPCAT1-mediated lipid reprograming and the TGF-β/Smad2/3 signaling pathway. We believe that our study provides more insights into the role of LPCAT1 in EC, and LPCAT1 may be a potential novel cancer therapeutic target for EC.

## Supplementary Data

Supplementary data is available at *Acta Biochimica et Biophysica Sinica* online.
